# A community health worker-led multimedia intervention to increase cervical cancer screening uptake among South Asian women: study protocol for a cluster randomized wait-list controlled trial

**DOI:** 10.1186/s13063-019-3378-4

**Published:** 2019-05-14

**Authors:** Cho Lee Wong, Winnie Kwok Wei So, Dorothy Ngo Sheung Chan, Kai Chow Choi, Tika Rana

**Affiliations:** The Nethersole School of Nursing, Faculty of Medicine, The Chinese University of Hong Kong, Room 824, Esther Lee Building, Hong Kong, People’s Republic of China

**Keywords:** Cervical cancer, Community health worker, Screening, Multimedia

## Abstract

**Background:**

Marked ethnic disparities on cervical cancer screening have been observed among South Asian women. Multiple barriers, such as language difficulties, poor access to screening services, values, and beliefs, were identified. Multimedia interventions led by community health workers (CHWs) would likely reduce screening disparities and increase cervical screening uptake among South Asian women. This study aims to assess the effects of a CHW-led multimedia intervention on the uptake of cervical cancer screening among South Asian women.

**Methods:**

This study is a cluster randomized wait-list controlled trial. A total of 408 South Asian women from Pakistan, India or Nepal will be recruited from six ethnic minority associations. Each association will be randomized to one of the two arms: an intervention arm (*n* = 3) that will undergo immediate treatment (CHW-led multimedia intervention) or a wait-list control arm (*n* = 3) that will receive delayed treatment. Each recruited CHW will be allocated to either arm according to the association she is affiliated with. The intervention arm will receive a CHW-led intervention comprising two components: multimedia education, and monthly telephone follow-up and navigation assistance. Participants in the control arm (*n* = 3) will be offered the CHW-led intervention after those in the intervention arm have completed the intervention. The primary outcome measure is the uptake of cervical cancer screening. Secondary outcomes include readiness to undergo screening and beliefs regarding cervical cancer screening. Outcomes assessments will be performed at baseline, immediately after, and 3 months after completion of the intervention.

**Discussion:**

The results of this study will potentially provide significant practical implications for addressing the needs and increasing the uptake of cervical cancer screening among South Asian women.

**Trial registration:**

Chinese Clinical Trial Registry, ChiCTR1800017227. Registered on 18 July 2018.

**Electronic supplementary material:**

The online version of this article (10.1186/s13063-019-3378-4) contains supplementary material, which is available to authorized users.

## Background

Cervical cancer, one of the most common cancers that affect women in Hong Kong, poses a considerable healthcare burden [1]. In 2015, 500 new cases of cervical cancer were diagnosed with a crude incidence rate of 12.7 per 100,000 among the female population [1]. A total of 151 women died from this cancer in Hong Kong in 2016 [1]. Cancer screening should be conducted to detect premalignant lesions and ensure that proper management is implemented early to prevent development, to reduce the burden of cervical cancer [2]. Early detection through cancer screening also enables the availability of several treatment options, thereby improving survival rates and reducing healthcare costs [2]. In Hong Kong, women aged 25–64 years who have sexual experience are recommended to undergo a Papanicolaou test (Pap test) once every 3 years after two consecutive annual Pap tests with normal results. Furthermore, women aged 65 years or older who never have a cervical smear should undergo a Pap test [2].

As the largest (14%) and one of the fastest growing ethnic minority groups in Hong Kong [3], South Asians from India, Nepal, and Pakistan represent an important target for health intervention. Studies on the rate of cervical cancer among South Asians living in Hong Kong are currently lacking. However, studies in western countries have consistently demonstrated that the incidence of cervical cancer among South Asian women is comparable to or even higher than that among the general population in these countries [4, 5]. Marked ethnic disparities in cervical cancer screening have also been observed [6, 7].

A previous study demonstrated that more than half of South Asian women aged 40 years or older in Hong Kong never have a Pap test [7]. Their screening rate is also significantly lower than that of the local general population [7]. Factors that contribute to their low screening uptake rate include health illiteracy, lack of knowledge about cervical cancer and the importance of its early detection, misconceptions about cancer and screening, poor access to screening services, and language barriers [6, 8, 9]. Strategies to increase the rate of screening uptake among this underprivileged group should be developed to reduce the morbidity and mortality contributed by screening disparities.

Various interventions have been demonstrated to enhance knowledge of cervical cancer and promote screening intentions and uptake among ethnic minority women. However, several systematic reviews and meta-analyses have suggested that effective interventions share a number of common characteristics. These include: being theory based; being culturally tailored; being composed of multiple intervention strategies; and being delivered in a community setting with assistance in scheduling/attendance in screenings [10–12]. A recent theory-based and culturally tailored multimedia educational intervention to promote cervical cancer screening among South Asians using a one-group pretest and posttest design has shown that 95.5% of 1061 South Asians participants agree that the intervention increases their knowledge on cervical cancer, 95.7% report understanding of the importance of screening, and 93.8% know where to go for screening [13]. Despite the positive effects of the multimedia intervention, focus group interviews have revealed that some participants experience difficulties in scheduling and attending health services for screening because of language and cultural barriers. Further limitations on studies reporting these interventions include the lack of randomization and evaluation of the rate of screening uptake by participants. Thus, strategies that enhance the participants’ access to screening services and address methodological limitations should be developed.

Strategies such as interventions led by community health workers (CHWs) may circumvent the aforementioned limitations and reduce screening disparities [14]. CHWs are lay individuals within a community, and they are trained as participants who link the members of the community with healthcare providers to facilitate intervention [15]. The advantage of CHW involvement in health-related interventions is their strong integration into the social and religious lives of their ethnic communities. They can be trained as culturally compatible role models to ensure that their involvement in the development and delivery of an intervention ensures cultural appropriateness of the intervention, can help provide support, and can reinforce healthy behaviors of the intervention participants [16]. The components of CHW-led interventions usually involve provision of health education and services, support, and navigation, such as helping their peers find their way through health systems to ensure timely screening [16].

CHW-led interventions have successfully motivated the use of cancer preventive services and enhanced access to these services among ethnic minority women [15, 16]. CHW-led interventions also offer a more cost-effective approach to increase cancer screening uptake than other interventions [17]. A systematic review has demonstrated that CHW-led interventions are effective in increasing screening utilization among participants who are ethnically concordant with the CHWs [18]. Another recent trial involving 560 women examining the effects of a CHW-led intervention has demonstrated that the self-reported utilization rate of the Pap test among participants in the intervention arm at 6 months was 13.3 times higher than that in the control arm (95% CI = 7.9, 22.3) [19]. However, this study is limited by the use of subjective measures to assess screening uptake.

To address the limitations of previous multimedia educational interventions [13] and CHW-led interventions, we propose to develop an educational intervention by adopting an innovative approach that combines the use of multimedia and CHWs in the intervention. We will then evaluate its effects in a randomized wait-list controlled trial. This approach will potentially provide significant practical implications in addressing the needs and increasing the uptake of cervical cancer screening of South Asian women. Ultimately, this study will help reduce screening disparities, enhance the awareness of South Asian women on the importance of cancer screening on cervical cancer prevention, and reduce the healthcare cost incurred by treatment and care of cervical cancer patients.

## Theoretical framework

The two theoretical frameworks used to guide the development of this intervention are the Health Belief Model (HBM) [20] and the PRECEDE–PROCEED framework [21]. The HBM consists of six factors that determine readiness for healthy behavioral change: perceived susceptibility to the disease, perceived severity of the disease, perceived benefits, perceived barriers to recommended action, self-efficacy in taking recommended action, and cues to the action on that recommendation [20]. The HBM has been widely used in behavior-related health research, and is also recommended as a conceptual framework for the development of health behavior interventions, including those concerning cancer prevention [20].

**Fig. 1:**
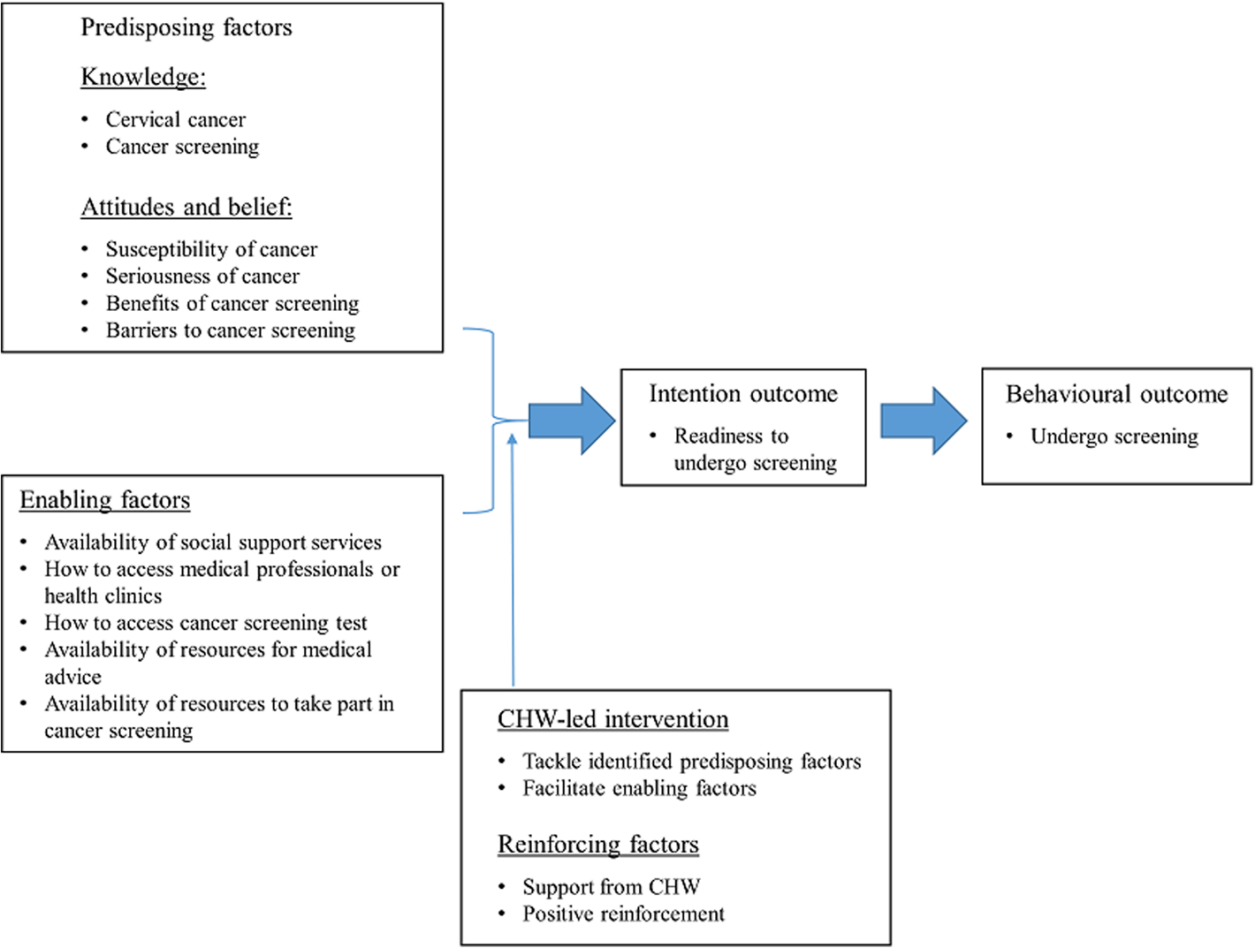
Theoretical framework to guide the development of the community health worker (CHW)-led intervention

The PRECEDE–PROCEED framework is a planning model for the development of behavioral change interventions. This framework emphasizes the needs of a target population and ensures that the intervention will be an accessible, acceptable, and effective means for working with ethnic minority groups. This framework allows the conceptualization of three main factors (predisposing, enabling, and reinforcing factors) which influence health behavior. The predisposition of factors, including knowledge, attitudes, and beliefs, influences an individual’s motivation for the target behavior. Enabling factors are those that empower an individual to act on motivation and accomplish the target behavior. Reinforcing factors promote the continuation of the particular behavior. Examples of such factors are reminders, positive reinforcement, and social support [22]. In this study, the HBM will be integrated into the PRECEDE portion of the PRECEDE–PROCEED framework to guide intervention development. These two frameworks can complement each other by focusing on health behavioral change and covering crucial factors that are likely to increase cancer screening uptake among ethnic minority women [22–24]. Figure 1 shows the theoretical framework.

## Objectives

The primary objective of this study is to evaluate the effects of a CHW-led multimedia intervention as measured by the uptake of cervical cancer screening among South Asian women immediately after and 3 months after completion of the intervention. The secondary objective is to assess the effects of such intervention on readiness to undergo screening and beliefs regarding cervical cancer screening (including perceived susceptibility to and seriousness of cancer, perceived benefits and barriers of cancer screening, and self-efficacy to undergo cancer screening) among those women.

## Hypothesis

Compared with the wait-list control arm, South Asian women in the intervention arm are expected to exhibit the following outcomes upon completion of the CHW-led multimedia intervention:

1. higher rates of cervical screening uptake evidenced by a record/receipt of screening utilization;
2. higher levels of readiness to undergo cervical cancer screening assessed by a question on readiness to undergo such screening; and
3. higher levels of perceived susceptibility to and seriousness of cervical cancer, perceived benefits of cancer screening, self-efficacy to undergo cancer screening, and lower levels in perceived barriers of cancer screening. (All items will be measured by the Cervical Cancer Screening Belief Scale.)

## Methods

### Design

This project has two phases. Phase I aims to recruit and train six CHWs who will lead the intervention in Phase II. Phase II is a cluster randomized wait-list controlled trial that aims to implement and evaluate the effects of the intervention. The rationale of using a wait-list control is to allow an untreated comparison for the intervention arm to determine whether the intervention has an effect. In addition, it allows the wait-listed participants who have never had a Pap test and have not undertaken cancer screening in the past 5 years to have the opportunity to receive future interventions.

**Fig. 2:**
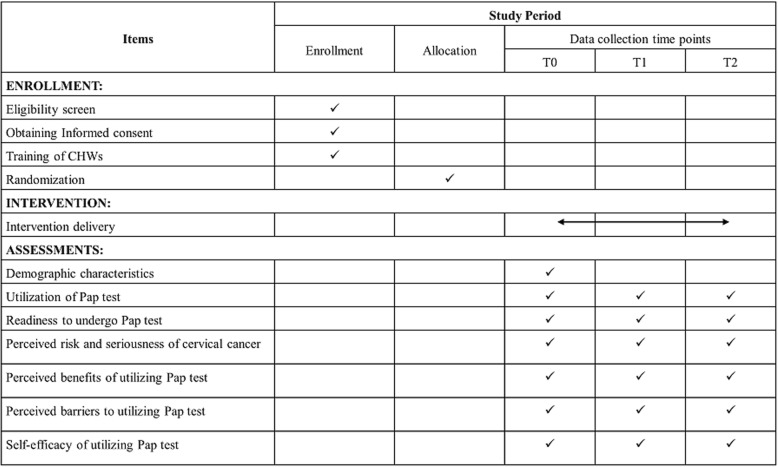
Standard Protocol Items: Recommendations for Interventional Trials (SPIRIT) figure. CHW community health worker, Pap Papanicolaou, T0 baseline (i.e., upon entry to the study after randomization but before participants receive the intervention), T1 immediate post intervention (3 months after T0, i.e., upon completion of the intervention), T2 3 months post intervention (6 months after T0, i.e., 3 months after completion of the intervention)

Standard Protocol Items: Recommendations for Intervention Trials (SPIRIT) 2013 (Additional file 1) will be used to report the study. The schedule for study enrollment, intervention, and outcome assessments is shown in Fig. 2 in the form of a SPIRIT diagram.

### Subjects

#### Subjects for Phase I: CHWs

Six CHWs (two Indians, two Nepali, and two Pakistani) will be recruited from six different community centers or ethnic minority associations in Hong Kong. These CHWs will be nominated by the persons in charge of these centers or associations involved in cancer prevention and women’s health among South Asians. A panel of research team members including three professors, one research nurse, and one research assistant with a medical background will select the most appropriate nominees. The potential CHWs should meet the following six criteria: able to demonstrate an understanding of South Asian women’s health needs and sociocultural norms; fluency in English and Urdu/Nepali/Punjabi/Hindi; willing to participate in community outreach activities and provide health information, healthcare, and social services relevant to cancer prevention; willing to complete the CHW training program and meet its requirements; and willing to serve as CHWs.

#### Subjects for Phase II: participants in the study

Prior to randomization, a trained research assistant (RA1) fluent in English and Urdu/Nepali/Punjabi/Hindi will approach potential subjects while they are attending various activities organized by the six participating community centers or ethnic minority associations. After an introductory presentation, women interested in participating in the study will be screened for eligibility. Inclusion criteria include: self-identified as South Asian (Pakistani, Indian, or Nepali); aged 25 years or older who have previously had sex; never had a Pap test or has not undertaken cancer screening in the past 5 years; have not been previously diagnosed with cancer; have not attended any intervention or education program related to cervical cancer screening in the previous year; and able to communicate in English, Urdu, Nepali, Punjabi, or Hindi and read English, Urdu, Nepali, or Punjabi.

### Sample size

The calculation of sample size is based on our previous survey on Hong Kong South Asians indicating that only 37% of them ever had a Pap test [7]. We anticipated that our intervention can increase the rate to at least 60%. By using power analysis software, PASS 14.0 (NCSS, Kaysville, UT, USA), it is estimated that 72 participants per arm will enable a two-parallel arm design with 80% power to detect a rate difference of at least 23% at a two-sided 5% level of significance, assuming the rate in the control is not greater than 37%. To allow for an attrition rate up to 15%, 85 participants in each arm will be needed. Furthermore, to account for potential reduction in statistical efficiency due to randomization by ethnic minority association (cluster) instead of individual participant, a variance inflation factor, called the design effect, is needed to impose on the sample size estimated [25]. The design effect is given by the equation: Design effect = 1 + (*m* – 1) × ICC, where *m* is the average cluster size and ICC is the intracluster correlation coefficient of the underlying outcome. A synthesis study revealed that the ICC tends to be small in primary care research, with a median of 0.005 and an interquartile range of 0.000–0.021 [26]. Allowing for an ICC of 0.021 in our proposed trial, at least 68 participants need to be recruited from each of the six ethnic minority associations—the number of participants in each cluster, *m*, is determined from the equation: number of participants = 2 × 85 × [1 + (*m* – 1) × 0.021] = 6 × *m*.

### Phase I: training of CHWs

A package of training materials was developed based on the existing literature and comments from the expert panel. The training program for the CHWs was examined in a previous feasibility study and shown to enhance participants’ knowledge of cancer, self-efficacy, and competence in working as CHWs [27]. The training of CHWs by the research team would require 14 h. Training will consist of seven sessions that cover the following: information about cervical cancer and screening; resources and access to screening tests; beliefs, myths, and misconceptions about cancer and cancer screening; barriers to cancer screening; facilitators and strategies to overcome such possible barriers; communication and problem-solving skills; and navigation support. The performance of the CHWs will be evaluated through an exit test. CHWs must obtain a pass in the exit test before they are considered eligible to deliver the CHW-led intervention.

### Phase II: implementation of the CHW-led intervention

#### Randomization and blinding

A cluster randomized trial is chosen because South Asian participants who are affiliated with the same center/association likely have close contact with one another through their attendance at the activities held at these centers or associations. Therefore, simple randomization will introduce a high risk of contamination because it can enable participants communicating the knowledge they have gained during the intervention with each other through their close contact, even though they are allocated to different arms.

Six centers and associations, located in districts with a large population of South Asians, have agreed to participate in this study. Each center or association will be randomized to one of the two arms: an intervention arm (*n* = 3) that will undergo immediate treatment (CHW-led multimedia intervention) or a wait-list control arm (*n* = 3) that will receive delayed treatment (the same CHW-led multimedia intervention, but provided after the intervention arm has completed the data collection). Each recruited CHW will be allocated to either arm according to the center or association she is affiliated with.

**Fig. 3:**
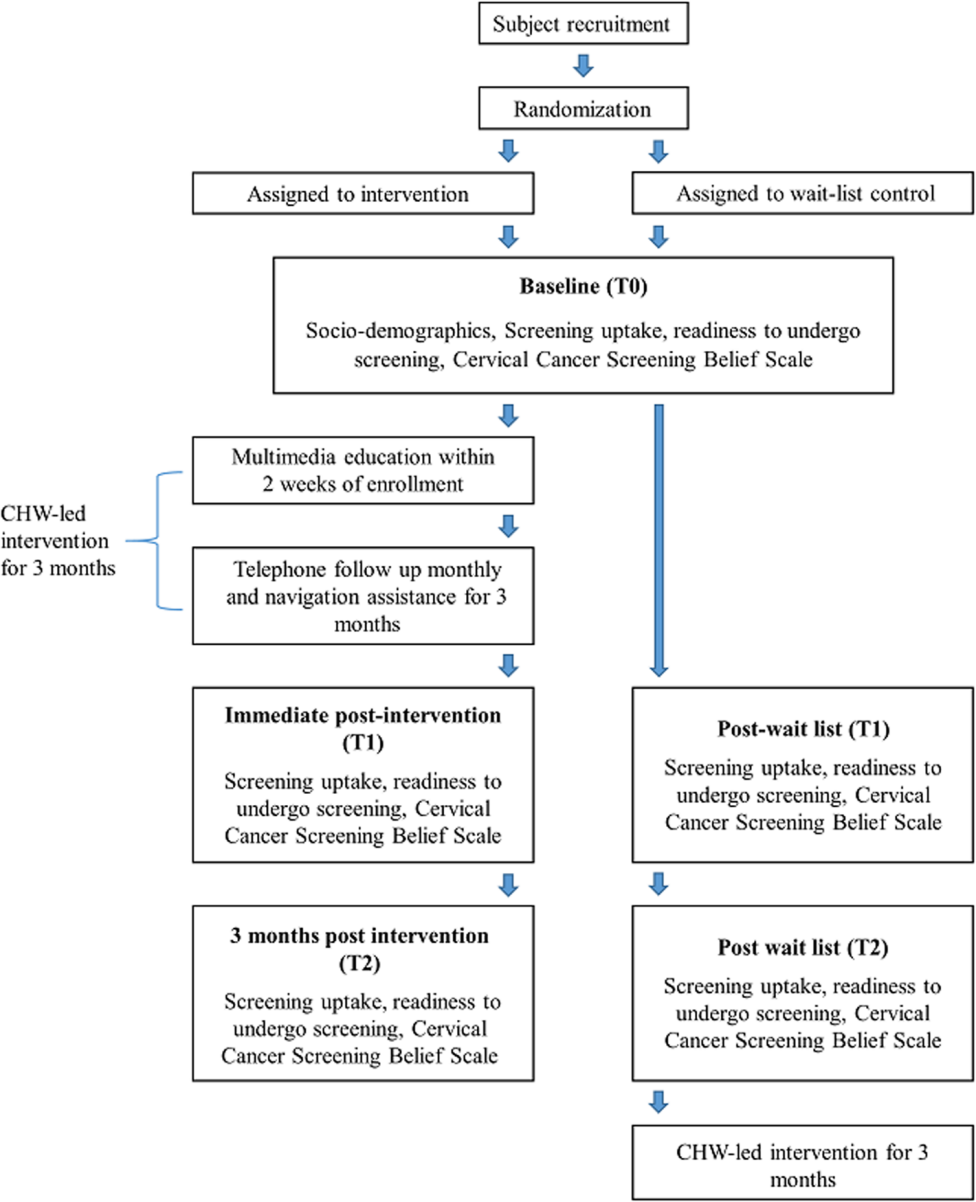
Flow diagram of the intervention and data collection points. CHW community health worker, T0 baseline (i.e., upon entry to the study after randomization but before participants receive the intervention), T1 immediate post intervention (3 months after T0, i.e., upon completion of the intervention), T2 3 months post intervention (6 months after T0, i.e., 3 months after completion of the intervention)

Research assistant 1 (RA1), who will help to recruit South Asian participants, will not know the subject allocation because recruitment is performed prior to randomization. A statistician, who is independent and blinded to the center/association and collection of data, will generate a random allocation sequence using a computer-generated randomization scheme. Participants from each center/association will be placed in their corresponding arm to avoid “contamination” across participants. Screening uptake and other secondary outcomes will be assessed by another research assistant (RA2) blinded to the arm allocation. CHWs will not be blinded because they are responsible for implementing the intervention. Figure 3 shows the study flow.

#### Fidelity of the intervention

The fidelity of the intervention will be ensured through the following means. First, at least one intervention conducted by each CHW will be randomly selected monthly to assess their compliance with the implementation protocol. Evaluations will be performed by the principal investigator. Second, the research team and the CHW will meet monthly to discuss the progress of the study.

### Interventions

#### CHW-led intervention arm

The CHW will contact the participants and schedule the intervention in the community centers or ethnic minority associations within 2 weeks after collection of the baseline data by the research assistant during study enrollment. Subjects in the intervention arm will receive the CHW-led intervention. The intervention has two components: multimedia education tested by our team members in an earlier study; and monthly telephone follow-up and navigation assistance.

This evidence-based multimedia educational intervention addresses the needs of South Asians by providing information on cervical cancer and detection methods, clarifying misconceptions, and providing information for access to various health services. The intervention design is consistent with the recommended features suggested in systematic reviews [10–12]: guided by the Health Belief Model; culturally relevant, linguistically appropriate, and written in common languages used by South Asians (English, Urdu, and Nepali); composed of multiple strategies (a 1-h health talk that uses a PowerPoint Presentation, a video clip, and a cervical cancer information booklet); and community based. Thus, strategies to enhance the participants’ motivation and facilitate access to screening services should be added to strengthen the intervention. Further studies should be conducted to address the methodological limitations.

The trained CHW will deliver two 30-min health talks using a PowerPoint presentation on the following topics: definition of cervical cancer and risk factors, signs, and symptoms of cervical cancer; myths and misconceptions about cervical cancer; preventive measures for cervical cancer; and available preventive measures and service providers in Hong Kong [27]. Participants will then be asked to watch a 4-min video clip to reinforce the knowledge gained. At the end of the health talk, participants will receive an information booklet about cervical cancer and information covered in the health talk. The CHW will discuss the contents of the booklet with the participants and instruct them on its use.

The telephone follow-up and navigation assistance will be conducted 3 months following the multimedia education. The CHWs will make a monthly telephone follow-up to reinforce the knowledge of participants and motivate them to undergo cervical cancer screening. CHWs will also offer navigation assistance, including making a screening appointment, accompanying participants to undergo screening, and completing screening-related paperwork.

#### Control arm

Subjects in the control arm will be offered the CHW-led intervention after the outcome data of the participants in both arms are collected.

### Outcome measures

#### Primary outcome

The primary outcome is the cervical screening uptake. Participants will be asked whether they have ever had a Pap test. A receipt of the screening test should be shown to the research assistant who will record the dates when the screening tests were undertaken.

#### Secondary outcomes

##### Readiness to undergo screening

Participants will be asked regarding their level of their readiness to undergo a Pap test within the next month.

##### Cervical cancer screening belief

The cervical cancer screening belief scale consists of 27 items in five scales: perceived susceptibility, seriousness, benefits, barriers, and self-efficacy of cervical cancer screening. Participants will be asked to respond to each item on a 5-point rating scale that ranges from “strongly agree (5)” to “strongly disagree (1)”. A high number selected in their responses would indicate a high level of perceived risk and seriousness of cervical cancer, greater perceived benefits and barriers of screening, and a high level of confidence to undertake screening tests. This scale has been translated into Nepali and Urdu, and tested among South Asian women in Hong Kong with good reliability and validity [28, 29].

##### Sociodemographics questionnaire

Age, ethnic group, monthly household income, years of formal education, marital status, employment status, number of years’ residence in Hong Kong, family history of cancer, and health insurance status of participants will be collected. Data on cues to action to screening (e.g., knowing where to go for screening) and any previous cervical screening experiences will also be collected.

### Data collection

Data for the primary and secondary outcomes (except for the sociodemographics questionnaire) will be collected by a research assistant blinded to arm allocation at three time points: baseline (T0), i.e., upon entry to the study after randomization but before participants receive the intervention; immediate post intervention (T1, 3 months after T0), i.e., upon completion of the intervention; and 3 months post intervention (T2, 6 months after T0), i.e., 3 months after completion of the intervention. The research assistant will contact the participants in both arms to complete the questionnaire. All participants will receive HK$30 upon completing the questionnaire to promote their retention and complete the follow-up.

No data monitoring committee is involved in this study because the intervention is unlikely to introduce a risk or an adverse event to participants.

### Statistical analysis

Data will be double-entered for validation and checked for range before analyses. Data will be summarized and presented using appropriate descriptive statistics. The normality of continuous variables will be assessed using skewness and kurtosis statistics and a normal probability plot. Appropriate transformations, if needed, will be made on skewed variables before entering them into the inferential analysis. The baseline characteristics of the intervention and control arms will be compared by chi-square or independent *t* tests as appropriate. The effectiveness of the CHW intervention will be evaluated by comparing the proportions of subjects in both arms reporting cervical cancer screening uptake at T2 and the changes in level of readiness to take cancer screening at T1 and T2 with respect to T0 between the two arms. The statistical significance of these outcome comparisons will be evaluated using a permutation test, accounting for the potential design effect of the randomization conducted at a cluster level instead of for individual participants [30]. Specifically, differences in uptake rates and changes in the level of readiness will be computed for all possible combinations of arm assignments of the six ethnic minority associations, and the 2.5 and 97.5 percentiles of these differences will be set as the critical values for two-sided permutation tests for the corresponding outcomes. Statistical analyses will also be conducted using SAS release 9.4 (SAS Institute Inc., Cary, NC, USA). All statistical tests will be two-sided with a level of significance set at 0.05.

### Ethical considerations

Ethical approval has been obtained from the ethical committees of the study institutions. Eligible participants will be briefed on the study details, its aims and objectives, and the rights of the subjects to participate or withdraw at any time. They will be given an assurance in terms of the anonymity and confidentiality before, during, and after the trial. Information collected about the subjects will be kept strictly confidential, and only the research team has access to the encrypted data file. All hard copies of documents will be kept in a locked cabinet and will be destroyed within 6 years of the study’s completion. The electronic data will be kept for 10 years. An information sheet with details of the study and an informed consent form will be provided. Potential subjects interested in the study will be asked to sign the consent form and return it to the CHWs. Voluntary participation will be emphasized among the participants, and they can leave the study at any time without giving a reason. Any protocol modifications will be communicated to the ethics committee and the trial sponsor and registered on the Chinese Clinical Trial Registry.

### Dissemination of results

Results of the trial will be communicated to participants after completion of the study upon request. Findings of the study will be submitted to peer-reviewed academic journals for publication.

## Discussion

To the best of our knowledge, this work is the first cluster randomized wait-list controlled trial to examine the effectiveness of a CHW-led multimedia intervention to increase cervical cancer screening uptake among South Asian women in Hong Kong. This evidence and theory-based intervention addresses the needs of South Asian women with an objective evaluation plan. This intervention is grounded in theoretical models, so findings will also demonstrate how such models can be integrated into the existing mainstream health services to enhance cancer screening uptake.

The combined use of multimedia CHWs in the intervention will potentially provide significant practical implications in addressing related barriers and increasing the uptake of cervical cancer screening among South Asian women. With the use of a multimedia approach in intervention delivery, information related to cervical cancer and cancer screening will be disseminated more effectively to South Asian women who generally have lower educational levels [13]. The navigation assistance provided by CHWs will further help these women overcome difficulties in accessing cervical cancer screening services. In addition, the target population includes hard-to-reach women who are rarely or never screened. Participation in the CHW-led intervention may help them establish their supporting networks and thus contribute positively to their community integration. Results will also be valuable for the government and policy-makers to maximize the community’s capacity to promote health through CHWs.

The strengths of this study include the use of objective measures in examining the uptake of cervical cancer screening by asking the participants to provide receipt of screening utilization. A previous study used a self-report approach to collecting data on cancer screening uptake [31]. However, blinding of participants and CHWs is not feasible in our study because of the nature of the intervention. Nevertheless, primary and secondary outcomes will be assessed by outcome assessors blinded to arm allocation to minimize potential bias of the results.

Certain challenges may be encountered throughout the study. First, the retention of CHWs during the training and implementation of the intervention may be challenging because South Asian women consider that family obligations should be prioritized over other activities, including those for health promotion [32]. In addition, their husbands or mothers-in-law play an influential role in their lives. When the CHWs become occupied by other family issues or their husbands do not allow them to take part in the study, we may need to recruit and train a new CHW to conduct the intervention. Second, the recruitment of South Asian women may be difficult because of the conservative nature of their culture [33]. Their religious beliefs may hamper their participation in this intervention.

To overcome these challenges, we will clearly explain the responsibility of the potential CHWs in this study. The training will be arranged at the time that is most convenient for these CHWs (typically when their children are attending school) and at the community centers located near their homes to facilitate their participation in the training program. We will also allow a longer time frame for the CHWs to complete the delivery of the interventions and thus minimize the disruption in their daily schedules during the study.

Furthermore, we will attempt to build up trust and use a direct approach to recruit potential participants. A South Asian research assistant, who is involved in various health promotion projects, will approach and recruit potential participants when they attend various social and religious activities. When the research assistant establishes a social relationship and rapport with the potential participants, she can easily raise their awareness of the importance of this project, likely facilitating the recruitment process. The familiarity and sensitivity of the research assistant to the cultural norms and religious practices among South Asians can also help alleviate the uncertainties that they may have about this study.

## Summary

South Asian women are significantly at a risk of developing cervical cancer, but their screening uptake rate is low. The proposed CHW-led multimedia intervention will potentially provide significant practical implications in addressing related needs and increasing the uptake of cervical cancer screening of this underprivileged group. The proposed intervention is self-sustainable and can be adopted by different organizations to reduce ethnic disparities on screening if the intervention is proven effective. Ultimately, this intervention may help reduce the healthcare burden posed by cervical cancer by increasing the awareness of South Asian women on the effective prevention of cervical cancer through early detection of the disease.

## Trial status

This trial commenced on 1 September 2018, and the anticipated end date of the study is 31 August 2020.

## Additional file

Additional file 1: SPIRIT 2013 Checklist: Recommended items to address in a clinical trial protocol and related documents (DOC 125 kb)

## Abbreviations

CHW: Community health worker
HBM: Health Belief Model
